# The role of the sex hormone-gut microbiome axis in tumor immunotherapy

**DOI:** 10.1080/19490976.2023.2185035

**Published:** 2023-03-07

**Authors:** Luoyang Wang, Lei Tang, Dongchang Zhai, Meiying Song, Wei Li, Shuo Xu, Suli Jiang, Haining Meng, Jie Liang, Yingying Wang, Bei Zhang

**Affiliations:** a Department of Immunology, School of Basic Medicine, Qingdao University, Qingdao, China; b Department of Special Medicine, School of Basic Medicine, Qingdao University, Qingdao, China; c School of Emergency Medicine, Qingdao University, Qingdao, China; d Shandong Provincial Key Laboratory of Animal Cell and Developmental Biology, School of Life Sciences, Shandong University, Qingdao, China

**Keywords:** Gut microbiome, sex hormones, immune checkpoint inhibitors, tumor immunotherapy

## Abstract

Accumulating evidence suggested that both gut microbiome and sex play a critical role in the efficacy of immune checkpoint blockade therapy. Considering the reciprocal relationship between sex hormones and gut microbiome, the sex hormone-gut microbiome axis may participate in the regulation of the response to immune checkpoint inhibitors (ICIs). In this review, it was attempted to summarize the current knowledge about the influences of both sex and gut microbiome on the antitumor efficacy of ICIs and describe the interaction between sex hormones and gut microbiome. Accordingly, this review discussed the potential of enhancing the antitumor efficacy of ICIs through regulating the levels of sex hormones through manipulation of gut microbiome. Collectively, this review provided reliable evidence concerning the role of the sex hormone-gut microbiome axis in tumor immunotherapy.

## Introduction

Through unleashing of antitumor T cell activity, immune checkpoint inhibitors (ICIs) have achieved great success in diverse types of cancer. However, some cancer patients have not responded to ICIs, indicating the necessity of searching for the reasons for the low responsiveness to ICIs^1^. Among the multiple potential factors, certain gut microbiome signatures have been found to be associated with a higher response to ICIs^2–4^. Both gut microbes and bacterial-derived metabolites may contribute to the influences of gut microbiome on the outcomes after receiving ICIs; however, the underlying mechanisms have not yet been fully clarified^5^.

Emerging evidence indicated the existence of the sex hormone-gut microbiome axis. The sex-based differences in gut microbiome have been extensively investigated. Changes in sex hormone levels induced by gonadectomy^6^, menstrual cycle and menopausal status^7,8^, or certain diseases (e.g., polycystic ovary syndrome (PCOS))^9^ can alter the gut microbial composition. In turn, transfer of gut microbiota^10^ or treatment with antibiotics^11^ resulted in significant changes in levels of sex hormones. The interaction between sex hormones and gut microbiome, termed the sex hormone-gut microbiome axis here, plays a vital role in multiple diseases, such as PCOS^9^, cardiovascular disease^12^, mental disorders^13^, etc.

Clinical meta-analysis indicated that the magnitude of benefit derived from ICIs is largely sex-dependent^14,15^. Moreover, sex hormone receptors were recently identified to play an important role in the sex-based immune response to ICIs via regulating CD8+ T cell function^16–19^. Considering the existence of the sex hormone-gut microbiome axis, gut microbiome may affect the antitumor efficacy of ICIs by regulating the host’s sex hormone levels. The present study aimed to review recent advances in understanding the underlying mechanisms by which gut microbiome and sex hormones could affect the immune response to ICIs. In particular, the interaction mechanisms between sex hormones and gut microbiome were explored, and the possibility of enhancing antitumor efficacy of ICIs via manipulating the sex hormone-gut microbiome axis was assessed.

## Gut microbiome could modulate the response to ICIs

The effects of gut microbiome on antitumor immunity were first revealed in mice with disruption of gut mucosal integrity induced by total body irradiation or cyclophosphamide^20,21^. The translocation of certain gut microbes, such as *Lactobacillus* and *Bifidobacterium*, into secondary lymphoid organs triggered by gut mucosal disruption could promote the activation of CD4+ or CD8+ T cells and improve the antitumor outcomes. Meanwhile, it was demonstrated that gut microbiome was necessary for the efficacy of cytosine-phosphate-guanine (CpG)-oligonucleotide-based tumor immunotherapy^22^. Then, the essential role of gut microbiome in ICI immunotherapy was investigated in anti-cytotoxic T-lymphocyte-associated protein 4 (anti-CTLA-4) or anti-programmed death-ligand 1 (anti-PD-L1) antibody-treated mice and the relevant bacterial species were identified by 16S ribosomal RNA sequencing^23,24^. It was found that the antitumor effects of CTLA-4 blockade depended on specific *Bacteroides* species^24^, while oral administration of *Bifidobacterium* facilitated anti-PD-L1 efficacy against melanoma^4^. These valuable findings from preclinical mouse models suggested a critical role of gut microbiome in cancer immunotherapy and inspired further investigation on human cancer patients. In 2018, three independent studies demonstrated that intestinal microbiome profoundly affected responses to anti-programmed cell death protein 1 (anti-PD-1)/anti-PD-L1 immunotherapy in patients with melanoma, non-small cell lung cancer (NSCLC), and renal cell carcinoma^2–4^. However, no consensus microbial signals associated with favorable response to ICIs were identified in these three studies, suggesting that function, rather than specific species, may better indicate the effects of intestinal microbiota^25^. Due to the limited concordance among different studies and the lack of understanding of the precise composition of a favorable gut microbiome, fecal microbiota transplantation (FMT) exhibited to be a promising method to eliminate resistance to ICI immunotherapy^26,27^. Multiple clinical trials have assessed the feasibility of improving the efficacy and safety of ICIs through FMT^28,29^. Two clinical trials have concentrated on the use of FMT to improve ICI response in patients with melanoma (Identifiers: NCT03341143 and NCT04988841), and one trial for metastatic castration-resistant prostate cancer (Identifier: NCT04116775) has moved to a phase II trial.

The influences of gut microbiome on the antitumor efficacy of ICIs may be mediated by multiple mechanisms (Table 1), which have not yet been fully explored^5^. Both gut microbes and their metabolites may contribute to the effects of gut microbiome on the cancer immune response. For instance, bacterial immunostimulants, such as peptidoglycan^30^, polysaccharide^24^, exopolysaccharide^31^, and type 1 fimbriae adhesion portion^32^, may enhance the antitumor effects of ICIs through strengthening both innate immunity and T cell functions in mice. Bacteria penetrating the mucus and submucosal lymphoid organs^21^ or even the tumor sites^33^ may also stimulate the antitumor immune response in mice. Besides, a cross-molecular mimicry may exist between bacteria and tumor-associated antigens, leading to the induction of cross-reactive CD8+ T cell responses^34^. Inspired by the similarity of gut microbiota and tumor-derived antigens, a therapeutic vaccine has been developed using “oncomimic” peptides in conjunction with anti-PD-1 for treatment of colorectal cancer (Identifier: NCT05350501).

**Table 1.** Possible mechanisms of the influences of gut microbiome on the antitumor efficacy of ICIs.

Effector component	Effect	Possible mechanism	Model organism	Ref.
**Bacterial immunostimulants or bacterial antigens**				
Peptidoglycan	Increase	Expression of peptidoglycan hydrolase promotes the generation of muropeptides, which can act as adjuvants through NOD2 receptor.	Mouse B16-F10 melanoma treated with anti-PD-L1, MCA205 fibrosarcoma treated with anti-PD-1, MC38 colorectal carcinoma treated with anti-CTLA-4.	30
Polysaccharide	Increase	*B. fragilis* polysaccharides trigger IL-12–dependent Th1 immune responses.	Mouse MCA205 sarcomas treated with anti-CTLA-4.	24
Exopolysaccharide	Increase	*Lactobacillus*-derived exopolysaccharides induce CCR6+ CD8+ T cells.	Mouse CCL20-expressing tumor model treated with anti-PD-1 and anti-CTLA-4 (Colon26 colon adenocarcinoma and 4T1 mammary carcinoma).	31
Type 1 fimbriae adhesion portion	Increase	Escherichia coli adhesion portion FimH induces TLR4-dependent DC maturation.	Mouse CT26 carcinoma treated with anti-PD-L1.	32
Gut bacterial antigens	Increase	Molecular mimicry between bacteria and tumor-associated antigens may induce cross-reacting CD8+ T cell responses.	Clinical trials of “oncomimic” peptides in combination with anti-PD-1 for treatment of colorectal cancer (NCT05350501).	34
**Bacterial metabolites**				
Butyrate	Increase	Boosting antitumor cytotoxic CD8+ T cell responses through IL-12 signaling pathway.	Mouse MC38 colon carcinoma treated with anti-PD-L1.	40
Butyrate and propionate	Decrease	Inhibiting anti-CTLA-4-induced DC maturation and T cell priming.	Mouse MC38 and CT26 colon carcinoma and MCA101_OVA_ ﬁbrosarcoma treated with anti-CTLA-4.	51
Inosine	Increase	Activating anti-tumor T cells through adenosine A_2A_ receptor; serving as an alternative carbon source for CD8+ T cells.	Mouse MC38 colon carcinoma treated with anti-CTLA-4 plus CpG, B16-F10 melanoma treated with anti-PD-L1.	41, 42
Kynurenine	Decrease	Unknown (higher kynurenine/tryptophan ratio is associated with resistance to anti-PD-1 treatment).	NSCLC patients were treated with anti-PD-1 antibodies.	50
Trimethylamine oxide (TMAO)	Increase	Induce tumor cell pyroptosis by activating the endoplasmic reticulum stress kinase PERK.	Mouse 66cl4 and 4T1 mammary carcinoma were treated with anti-PD-1.	43
Anacardic acids	Increase	Unknown (higher levels of anacardic acids in ICI responders).	Metastatic melanoma patients.	44

In addition to the immunomodulatory microbial components, the effects of gut microbiome on the antitumor immune response to ICIs can be mediated by their metabolites. Short-chain fatty acids^35–37^, the major end-products of gut microbiota-derived metabolites, and some short-chain fatty acid-producing bacterial species, such as *Akkermansia muciniphila* ^3^, *Lachnospiraceae* ^38^, and *Lactobacillus* ^39^, have been found to be associated with favorable response to ICIs in cancer patients. Particularly, butyrate could directly enhance the antitumor cytotoxic CD8+ T cell responses by promoting IL-12 signaling pathway and increase the efficacy of anti-PD-L1 therapy in mice^40^. Similarly, the purine metabolite inosine produced by certain bacteria, such as *Bifidobacterium pseudolongum* and *Akkermansia muciniphila*, could improve response to ICIs in mouse models of cancer via activating anti-tumor T cells through adenosine A_2A_ receptor^41^. Besides, inosine could serve as an alternative carbon source for CD8+ T cell function under glucose restriction and relieve tumor-imposed metabolic restrictions on T cells^42^. Supplementation with inosine enhanced the anti-tumor efficacy of ICIs in mice^41,42^. Another microbial metabolite correlated with efficacy of ICIs is trimethylamine oxide (TMAO)^43^. Choline or carnitine in the food can be transformed into the precursor trimethylamine by gut microbiome, which may be catalyzed to generate TMAO in the liver. Either intratumoral injection of TMAO or oral supplement with choline enhanced antitumor activity of anti-PD-1 in mice with triple-negative breast cancer^43^. Other gut microbiota-derived metabolites, such as anacardic acids^44^ and secondary bile acids^45^, may also affect the antitumor effects of ICIs. However, it is noteworthy that both TMAO and secondary bile acids may also promote carcinogenesis, especially in the colon^46–48^. In addition, higher plasma levels of TMAO and precursors may increase the risk of coronary heart disease^49^.

Some microbial metabolites may also decrease the antitumor efficacy of ICIs. For instance, the tryptophan released from degradation of dietary proteins can be converted into various metabolites by gut microbiome, such as indole, indole-related compounds, and kynurenine. As a typical immunosuppressive tryptophan metabolite, a higher kynurenine/tryptophan ratio has been found to be associated with a poor response to anti-PD-1 in NSCLC patients^50^. Moreover, the influences of microbial metabolites on anti-tumor immunity could be largely situation-dependent. For instance, high blood butyrate and propionate levels were reported to be associated with resistance to CTLA-4 blockade in patients with metastatic melanoma, and oral administration of sodium butyrate diminished antitumor efficacy of anti-CTLA-4 in mice^51^. It could be attributed to the difference between anti-CTLA-4 and anti-PD-1/PD-L1, which needs to be validated in the future. In addition, considering the large pool size of gut microbiota-derived metabolites, the causal relationship for most of the metabolites should be further confirmed, although a strong correlation may be identified.

Of note, ICI immunotherapy can also alter the composition of the gut microbiome. It has been shown that there is a variation in the gut microbiome composition during anti-PD-1 immunotherapy in patients with hepatocellular carcinoma^38^. The effects of ICIs on gut microbiome were also observed in an animal study^24^. Furthermore, our previous study indicated that anti-PD-L1 could significantly alter the composition of the gut microbiome and decrease the relative abundance of *Lachnospiraceae* in female mice while exerting no effect on male mice^52^. The underlying mechanisms of the impact of ICIs on gut microbiome have yet to be comprehensively clarified. The activation of T cells in the intestine could be associated with these effects^24^.

## Sex-based differences in response to ICIs

Various factors have been found to be associated with antitumor response to immunotherapy, including tumor-cell-intrinsic features (e.g., PD-L1 expression, tumor-associated antigens, tumor burden, tumor mutational burden, mismatch repair deficiency, and epigenetic alterations), tumor immune microenvironment signatures (e.g., tumor-infiltration lymphocytes and the presence of immunosuppressive cells), and environmental factors, such as diets and intake of antibiotics^1,53–55^. However, recognition of the importance of sex on ICI response is relatively recent. Sex-based differences in tumor incidence and mortality are evident for most types of cancer, in which male patients have higher incidence rates ranging from 1.26:1 to 4.86:1^56^. These differences may be partially attributed to sex-based differences in the immune system. Generally, women have stronger innate and adaptive immunity, higher incidence rates of autoimmune diseases, better vaccine responses, and greater tolerability of adverse effects of vaccination^57,58^. Evidence from meta-analysis of clinical trials mainly indicated that men responded better to ICIs than women^14,59–62^. However, this trend has not always been confirmed. A trend of higher response rate in female patients compared with male patients with NSCLC was also reported^63^, which is contradictory to other studies^14,60,61^. In contrast, one meta-analysis included 23 randomized clinical trials (9322 men and 4399 women) demonstrated no statistically significant difference in response to ICI immunotherapy between the sexes^64^. Moreover, female patients with advanced lung cancer achieved a significantly greater benefit from the combined therapy of chemotherapy and anti-PD-1/PD-L1, whereas male patients responded better to anti-PD-1 alone^15^. Thus, addressing these concerns by meta-analysis pooling different clinical trials may not be adequate^63^. The relatively large fluctuation of levels of sex hormones among patients due to either physiological or pathological factors may partially account for the discrepancy. Besides, our previous studies on mice revealed that anti-PD-L1 treatment could significantly affect male sex hormone levels, further complicating our understanding of the role of sex in tumor immunotherapy. Notwithstanding, signiﬁcantly higher tumor mutational burden, single-nucleotide variation neoantigen load, and PD-L1 expression level could be found in male patients with melanoma^63,65^. In addition, studies showed that androgen deprivation therapy could significantly enhance the antitumor efficacy of ICIs in mice^52,66^. Although whether there is a sex-based difference in survival benefits from ICIs remains controversial, which may be addressed in future clinical studies covering more types of cancer other than melanoma and lung cancer, the effects of sex-relevant features on the antitumor immunotherapy deserve oncologists’ attention.

Many factors including hormones, genetic differences, and environmental factors are involved in the formation of sex disparities^67,68^. Although sex hormones cannot account for all sex-based differences in cancer^56^, they play a critical role in anticancer immunity^68^. Sex hormone receptors are nearly expressed in all immune cells, which participate in the regulation of the expression levels of many immune-related genes^68^. In general, estrogen enhances both innate and adaptive immunity, while androgen suppresses immune cell activity^58^. However, most studies demonstrated that male patients tend to achieve greater survival benefits from ICI immunotherapy, which seems to be inconsistent with the effects of sex hormones. The sex-based immune features, such as tumor mutational burden and tumor infiltration of immune cells, may contribute to the differences in the efficiency of immunotherapy between male and female patients^63^. On the other hand, our previous study showed that anti-PD-L1 could significantly downregulate the levels of sex hormones in male mice rather than in female mice, which enhanced the antitumor efficacy of anti-PD-L1^52^. In contrast, Tulchiner *et al*. reported that anti-PD-1 immunotherapy significantly increased estradiol and luteinizing hormone (LH)/follicle-stimulating hormone (FSH) ratio in male patients with metastatic renal cell carcinoma from the beginning of therapy to week 12 of follow-up, while it had no influence on testosterone level in both sexes^69^. The potential effects of ICIs on the levels of sex hormones and in turn their impacts on the sex-based differences in the response to ICIs have not yet been fully explored and deserve further investigation.

## Sex-based differences in gut microbiome

A growing body of evidence from both human and animal studies indicated the existence of sex-based differences in microbiota composition^70–74^. Generally, women tend to have a higher alpha diversity. In a study of 1135 individuals from a population-based Netherlands cohort, women had a greater microbial diversity based on the values of the Shannon index^6^. Similarly, one study involving 551 healthy Chinese participants also revealed that women had a significantly higher alpha diversity in the fecal microbiota as assessed by the observed number of operational taxonomic units and the values of the Shannon index^75^. In another study from Italy, a significant increase in the Chao1 index and Shannon index was found in the mucosa-associated microbiota of female participants sampled by sigmoid brush^76^. Besides, a significantly higher species richness estimated by the Chao1 index was also found in wild-type female mice compared to male mice^73^. *Firmicutes* and *Bacteroidetes* are two of the most primary bacteria identified in the gut of both humans and animals, and the ratio of *Firmicutes* to *Bacteroidetes* (F/B ratio) is a widely used marker for gut dysbiosis, which has been associated with a number of health status-related factors, such as hypertension^77^ and obesity^78^. The F/B ratio also tends to be higher in women. For instance, one recent study from Ukraine involving 2301 healthy participants revealed that the F/B ratio was significantly higher in women than in men^79^. A higher F/B ratio was also found in healthy post-menopausal women than in pre-menopausal women or their corresponding age-matched men^7^. In addition, a lower abundance of *Bacteroidetes* was identified in women from a cohort conducted in the United States^80^. Notably, the alpha diversity of gut microbiome has been reported to be strongly negatively correlated with the relative abundance of *Bacteroidetes* ^81,82^. The higher microbial alpha diversity in females may be attributed to their relatively lower abundance of *Bacteroidetes*. However, the F/B ratio significantly varies among individuals and is noticeably affected by geographical latitude^82,83^, and no significant effect of sex on the F/B ratio has been found in some studies, possibly due to the large geographic scale or limited sample size^83,84^. Besides, it is noteworthy that there are also sex-based differences in the gut microbiome in cancer patients^85^. Moreover, there are distinct changes in microbial alpha diversity and community composition between the sexes during the development of colorectal cancer^85^. These sex-based differences in gut microbiome have been suggested to contribute to the sex-based disparity in liver carcinogenesis in mice^48^. The role of the sex-based differences in gut microbiome during cancer development and progression deserves further assessment.

Although summarizing the sex-based differentially abundant bacterial taxa is a challenge due to the lack of consistency among studies, some sex hormones-associated bacteria have been identified^86,87^. For instance, total levels of urinary estrogen in men and postmenopausal women were significantly associated with fecal *Clostridia* ^88^. The ratio of urinary estrogen metabolites to parent estrogen (estrone and estradiol) was positively correlated with the relative abundance of *Clostridiales* in healthy postmenopausal women, while it was inversely associated with the genus *Bacteroides* ^89^. The abundance of *Acinetobacter*, *Dorea*, *Ruminococcus*, and *Megamonas* was signiﬁcantly positively correlated with serum testosterone levels in men, while the abundance of *Slackia* and *Butyricimonas* was negatively correlated with serum estradiol levels in women^84^. In addition, studies demonstrated that sex hormones play an essential role in shaping the host gut microbiome. Changes in levels of sex hormones induced by drug administration (e.g., oral contraceptives)^8^, gonadectomy^6^, menstrual cycle, and menopausal status^7,8^, or certain diseases, such as polycystic ovary syndrome^9^, could alter the diversity or composition of gut microbiome. Animal studies further confirmed the effects of sex hormones on gut microbiome^90–93^.

Although the underlying mechanisms remain elusive, several potential pathways may be involved in the regulation of gut microbiome by sex hormones. Firstly, bacteria that express β-glucuronidase enzyme can release free sex hormone molecules from the conjugated metabolites that previously formed in the liver, which may result in the liberation of the glucuronic acid group and produce energy for gut bacteria^9^. Secondly, as mentioned earlier, sex hormone receptors are widely expressed in immune cells^68;^ thus, sex hormones may influence gut microbiome by regulating intestinal immune response. Knock-out of estrogen receptor β (ERβ) in female mice could result in significant changes in the composition of gut microbiome^92^. Besides, animal studies revealed substantial sex-based differences in IgA level^71^ and the expression levels of anti-microbial peptides^94^. These findings demonstrated that intestinal immunity might contribute to the regulation of sex hormones on gut microbiome. More recently, Nuriel-Ohayon *et al*. reported that progesterone supplementation increases the abundance of *Biﬁdobacterium* in mice and *in vitro* ^95^, indicating the favorable metabolism of sex hormones by certain bacterial taxa may also alter gut microbial composition.

## Gut microbiome could participate in regulation of sex hormone levels

The concept of “microgenderome” has been used to describe the bidirectional interaction between sex hormones and gut microbiome^96^. Sex-specific microbiome profiles that emerged after puberty contribute to the levels of sex hormones, which affect the development of autoimmune disease in the non-obese diabetic mouse model of type 1 diabetes (T1D)^10^. Elevating testosterone levels by transferring gut microbiota from adult male mice to immature female mice reduced T1D incidence. Furthermore, only particular microbes, such as segmented ﬁlamentous bacteria and a proteobacterium isolated from male mice, have been correlated with serum testosterone concentration and colonization with these bacteria conferred the protection against T1D in male mice^97^. These findings indicate that some gut microbes can participate in the regulation of the levels of sex hormones and ultimately modify the host’s autoimmunity.

**Figure 1.**
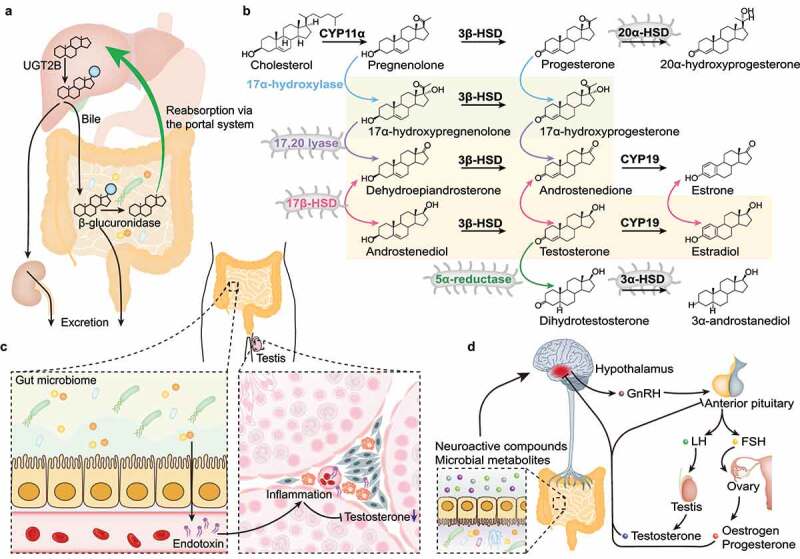
Potential mechanisms by which gut microbiome may participate in regulating the host’s sex hormone levels. (A) the glucuronidation of sex hormones catalyzed by uridine diphosphate-glucuronosyltransferase 2B (UGT2B) in the liver increases the water solubility, which promotes the excretion of the glucuronidated compounds via urine or bile to the small intestine. Part of the conjugated sex hormones are de-conjugated by β-glucuronidase from the commensal gut bacteria. After deconjugation, the free sex hormone molecules are reabsorbed via the portal system. (B) Certain bacterial enzymes, such as 3α-HSD, 17β-HSD, 20α-HSD, 5α-reductase, and 1720 lyase, may participate in the biosynthesis of steroid hormones in the intestine, whereas further investigation is required. (C) Some bacteria are important for the maintenance of the protective function of the mucus. Destruction of the intestinal barrier may facilitate the passage of gut bacteria into the systemic circulation and elicit a chronic state of inflammation, which may impair testicular function, including the testosterone production by Leydig cells. (D) Some gut microbes are involved in the metabolism of neuroactive compounds or regulation of the gut-brain mediator secretion, which may influence the activity of the central nervous system via the gut–brain axis. Gut microbiome may thus influence endogenous production of sex hormones via the hypothalamic–pituitary–gonadal axis.

Several potential mechanisms may be involved in the regulation of the host’s levels of sex hormones by gut microbiome (Figure 1). Firstly, gut microbes may participate in the metabolism of sex hormones in intestine through expressing certain enzymes. After glucuronidation mainly in the liver, the glucuronidated steroid sex hormones are excreted in urine or via biliary excretion to intestine^98^. These conjugated sex hormones are re-absorbed via enterohepatic circulation after deconjugation by β-glucuronidase from intestinal bacteria. It has been found that there is a noticeable free androgen in the intestine, which is 70-fold higher than that in the serum of young adult men^98^. Thus, gut microbiome may play an important role in regulating circulating androgen or estrogen levels through deconjugation by β-glucuronidase in intestine^98,99^. Totally, 279 unique microbiome-encoded β-glucuronidase proteins clustered into six unique structural categories have been identified in the Human Microbiome Project database^100^, of which certain members within three classes could reactivate estrogens from their inactive glucuronide forms^101^. Similarly, bacterial β-glucosidases and sulfatases may also participate in the deconjugation of conjugated sex hormone metabolites in intestine^87,102^. Importantly, human fecal bacteria can carry out a variety of reductive, oxidative, and hydrolytic reactions of androgens and estrogens^102^. The typical steroid-metabolizing enzymes involved in bacterial metabolism of sex hormones include hydroxysteroid dehydrogenase (HSD) and steroid reductase^103^. For instance, *Streptococcus mutans*, which showed a higher abundance in the gut microbiome from steroid inhaler users in patients with irritable bowel syndrome^104^, has been identified with the potential of metabolizing progesterone and testosterone by expressing 5α- and 5β-steroid reductases and 3α-, 17β-, and 20α-HSDs^105^. More recently, evidence supported the existence of bacterial enzymes (with a high sequence homology to human 1720 lyase) that would be responsible for the androgenic steroid biosynthesis in specific intestine microbiota species, such as *Ruminococcus gnavus*, contributing to endocrine resistance in castration-resistant prostate cancer^11^.

Secondly, gut microbes may also directly affect gonadal function. Some mucus-degrading bacteria (e.g., *A. muciniphila*) are essential for the maintenance of the protective function of the mucus^106,107^. Destruction of the intestinal mucosal barrier may facilitate translocation of gut bacteria from gut lumen into circulation, triggering systemic inflammation that inhibits production of testosterone by Leydig cells^108^. In healthy men, low-dose endotoxin challenge produced an acute systemic inﬂammatory response, followed by a significant decline in plasma testosterone levels, without influencing LH or FSH^109^. Similarly, our previous findings revealed that anti-PD-L1 could significantly downregulate the testosterone levels in male mice without affecting LH or FSH levels, probably through inducing local or systemic inﬂammatory response^52^. Moreover, oral supplementation with *Lactobacillus* could significantly reduce diethylhexyl phthalate-induced increase in the serum lipopolysaccharide level and recover the testosterone concentration in male mice^110^. All these evidences support that gut microbiome may participate in the regulation of the testis testosterone secretion via regulating inﬂammation. In addition, the microbiota-derived gut-brain mediators may influence gonadal hormone secretion through the gut-brain axis. In PCOS patients, oral administration of probiotic *Biﬁdobacterium lactis* V9 significantly promoted the secretion of gut-brain mediators, including ghrelin and peptide YY, decreased the ratio of LH/FSH, and increased the levels of sex hormones, demonstrating the possible involvement of the gut-brain axis^111^. Collectively, gut microbiome may take part in regulation of the host’s sex hormone levels via a variety of pathways, in which the detailed mechanisms have not yet been fully explored.

## Modulating levels of sex hormones via manipulation of gut microbiome to enhance antitumor efficacy of ICIs

Emerging evidence suggests that the sex hormone receptor signaling pathway is involved in modulation of CD8+ T cell function. Androgen receptor (AR) signaling could promote the transition from stem cell-like CD8+ T cells to terminally exhausted CD8+ T cells in male mice, and it was correlated with tumor-inﬁltrating CD8+ T cell exhaustion in cancer patients^16^. ERβ augmented the downstream TCR signaling cascade, and the combined use of ERβ agonist and anti-PD-1 substantially increased tumor-infiltrating CD8+ T cells and sensitized various syngeneic tumors to ICI immunotherapy in mice^18^. In contrast, ERα could promote macrophage polarization toward an immune-suppressive state, leading to CD8+ T cell dysfunction and exhaustion^19^. Inhibition of ERα using the selective estrogen receptor significantly increased the antitumor efficacy of ICIs in mouse models of melanoma^19^. Taken together, it is a feasible approach to improve the response to ICI therapy through regulating the sex hormone receptor signaling pathway.

To date, trials that investigated the potency of the possible combination of sex hormone intervention and tumor immunotherapy mainly concentrated on treatment of prostate cancer and breast cancer^112^. For instance, the combination of enzalutamide and a cancer vaccine significantly improved the overall survival rate in the TRAMP mouse spontaneous prostate cancer model^113^. In the semi – hormone-dependent Myc-CaP mouse tumor model, combining CpG and surgical orchiectomy or abiraterone reduced tumor burden and more effectively delayed tumor relapse than either single treatment^66^. Guan *et al*. reported that the AR expressed on CD8+ T cells could repress IFN-γ expression level and mediate the resistance to ICI therapy^17^. Surgical orchiectomy plus enzalutamide with anti-PD-L1 antibodies led to significant tumor regression and increased the overall survival rate in either an androgen deprivation therapy plus anti-PD-1-resistant mouse prostate tumor model or an AR-negative mouse sarcoma tumor model^17^. Besides, multiple ongoing clinical trials are investigating the efficiency of ICIs in combination with anti-estrogen therapy for breast cancer^114^. More recently, it was found that a combination of 17β-estradiol and anti-PD-L1 significantly inhibited MC38 colon tumor growth in male mice^115^. The findings mentioned above confirmed the feasibility of improvement of the efficacy of ICIs by modulating the levels of sex hormones. However, it is noteworthy that most existing trials have concentrated on improving the treatment of sex hormone-dependent cancer. It is essential to clarify whether the combination therapy is superior to ICI monotherapy for non-sex hormone-dependent cancer considering the potential side effects of sex hormone therapy. Furthermore, some nonsteroidal AR antagonists, such as flutamide and enzalutamide, have been shown to inhibit early-phase T cell activation and suppress the antitumor efficacy of anti-PD-L1 in mice^66^.

**Figure 2.**
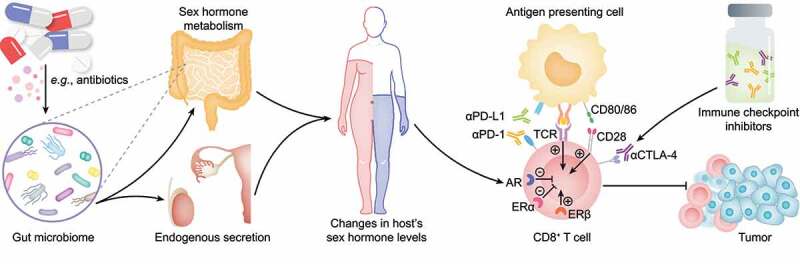
Modulating the levels of sex hormones via manipulating gut microbiome to facilitate antitumor efficacy of ICIs. Drugs, such as antibiotics, can alter the composition of gut microbiome and induce changes in the host’s sex hormone levels by interfering with their microbial metabolism in the intestine or regulating the endogenous production via the gut-brain axis or inflammation. Activation of AR or ERα signaling pathway may attenuate the antitumor efficacy of ICIs by inducing CD8+ T cell exhaustion, while ERβ may augment the downstream TCR signaling cascade and increase tumor-infiltrating CD8+ T cells. Thus, the antitumor efficacy of ICIs may be improved using drugs, such as antibiotics, to alter the gut microbiome to regulate the host’s levels of sex hormones. However, it is noteworthy that administration of broad-spectrum antibiotics may impair the antitumor efficacy of ICIs.

Recently, *Pernigoni* et al. reported that the commensal gut microbiome contributes to endocrine resistance in castration-resistant prostate cancer by providing an alternative source of androgens^11^. Importantly, ablation of the gut microbiome with a cocktail of broad-spectrum antibiotics delayed the emergence of castration resistance in both TRAMP-C1 allograft and the Ptenpc^–/–^ prostate conditional mouse models^11^. These results provide a novel approach to regulate the levels of sex hormones via manipulation of gut microbiome, which may be appropriate for the adjuvant therapy of non-sex hormone-dependent cancer. Given the gut microbiome’s great volatility, manipulating gut microbiome’s function with antibiotics rather than maintaining a specific bacterial species composition is a more reliable approach. However, numerous clinical studies have shown a detrimental effect of broad-spectrum antibiotics on ICI therapy, while the underlying mechanisms have been poorly explored^53^. In contrast, our previous study showed that oral administration of colistin, a narrow-spectrum antibiotic, could significantly downregulate the testosterone level in male mice and enhance the antitumor efficacy of anti-PD-L1 antibodies^52^. These findings confirmed the feasibility of improving the efficacy of antitumor immunotherapy by modulating levels of sex hormones via manipulation of the gut microbiome with narrow-spectrum antibiotics (Figure 2). Other commonly used methods to modulate gut microbiome, including FMT, probiotics, and prebiotics have also been tested for improving antitumor efficiency of ICI immunotherapy^26,27,116–118^, which can also affect the host’s levels of sex hormones^10,111,119^. However, it remains elusive whether sex hormones play a role in this process. Besides, additional studies are needed to clarify whether there is a synergistic effect of sex hormones and other microbial metabolites on ICI therapy.

## Conclusions

**Figure 3.**
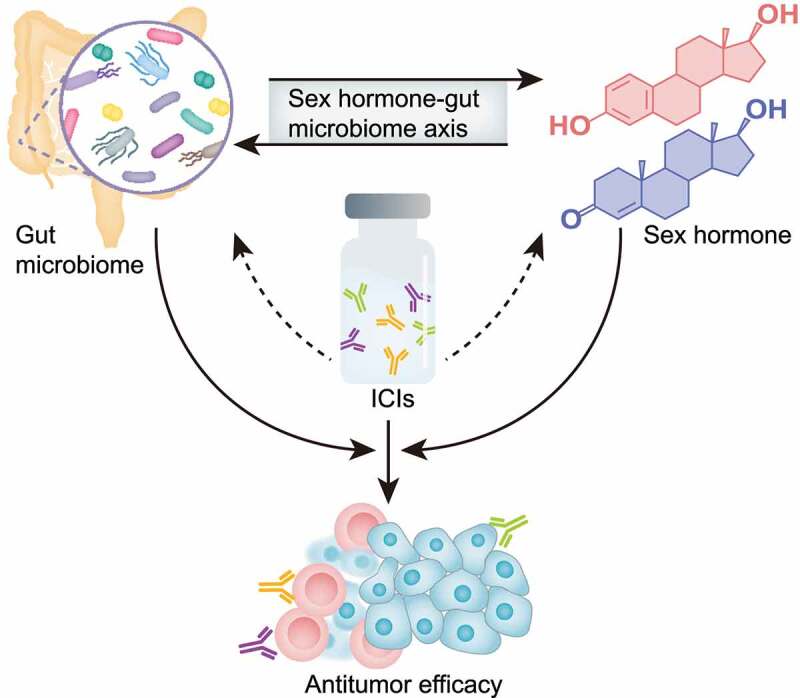
The role of the sex hormone-gut microbiome axis in tumor immunotherapy. Gut microbiome can influence the host’s levels of sex hormones through either metabolizing sex hormones or regulating gonadal secretion. In turn, the sex hormones can alter the gut microbiome by either serving as an energy source to support the growth of certain bacteria or regulating intestinal immune homeostasis. The interaction between sex hormones and gut microbiome constitutes the sex hormone-gut microbiome axis. Thus, the influences of gut microbiome and sex hormones on the patient’s response to ICIs can be simultaneously studied, although a growing body of evidence showed the effect of each of them on the antitumor efficacy of ICIs.

In conclusion, a growing body of evidence demonstrated that the sex hormone-gut microbiome axis might be involved in regulating the antitumor efficacy of ICIs (Figure 3). However, the underlying mechanisms have not yet been fully elucidated. Moreover, the potential effects of ICIs on the patient’s levels of sex hormones and the gut microbiome further complicate the role of the sex hormone-gut microbiome axis in ICI immunotherapy. Further research is required to elucidate the underlying mechanisms of the gut microbiome-mediated regulation of levels of sex hormones and develop new methods to manipulate the levels of sex hormones via targeting gut microbiome. Besides, the importance of the sex hormone-gut microbiome axis in tumor immunotherapy should be further evaluated considering the fact that several other gut microbial metabolites have also been found to be associated with the patient’s response to ICIs. Altogether, the sex hormone-gut microbiome axis provides a promising target for improving antitumor efficacy of ICI immunotherapy.

## Data Availability

Data sharing is not applicable to this article as no new data were created or analyzed in this study.
